# Can Creeping Fat Be a Prognostic Factor in Crohn’s Disease?

**DOI:** 10.5152/tjg.2025.25259

**Published:** 2025-11-12

**Authors:** Naime Demiral, Fatma Ayça Edis Özdemir, Fatih Kıvrakoğlu, Öykü Tayfur Yürekli, Fatma Ebru Akın, Mustafa Tahtacı, Fırathan Sarıaltın

**Affiliations:** 1 Department of Internal Medicine, Ankara Bilkent City Hospital, Ankara, Türkiye; 2 Department of Radiology, Ankara Bilkent City Hospital, Ankara, Türkiye; 3 Department of Gastroenterology, Osmaniye State Hospital, Ankara, Türkiye; 4 Department of Gastroenterology, Ankara Yıldırım Beyazıt University Faculty of Medicine, Ankara, Türkiye

**Keywords:** Adipose tissue, Crohn’s disease, obesity, stricture, subcutaneous fat, visceral fat

## Abstract

**Background/Aims:**

The aim of this study is to investigate whether the creeping fat (CF) finding evaluated by computed tomography (CT) can be used as a noninvasive marker to predict disease prognosis.

**Materials and Methods:**

The study comprised 128 Crohn’s patients, aged 18-70, who had CT scanning for a variety of reasons. Patients were retrospectively analyzed in 3 groups (31 patients with operation due to stenosis, 39 patients with unoperated stenosis, and 44 patients without stenosis). The CF Index BSACR (bowel surface area coverage ratio), CF density, and visceral-subcutaneous fat ratio (VSFR) findings were evaluated by CT compared between the groups.

**Results:**

The median VSFR was higher in the stenosed group compared to the non-stenosed group (0.7 vs 0.4; *P* = .003). The CF index BSACR was 37.5% (27.1% vs 18.2%; *P* = .001), and the rate of those with a CF index BSACR above 37.5% was higher in those with stenosis compared to those without. The odds of stenosis were 17.07 times (odds ratio [OR] = 17.07; *P* = .007) higher in those with a CF index BSACR of 37.5% and 184.57 times (OR = 184.57; *P* = .001) higher in those with a CF index BSACR of 50% or more compared to those with no involvement or a CF index BSACR of 25% or less.

**Conclusion:**

The CF index correlated with stricturing disease and may be a noninvasive radiologic marker that predicts disease prognosis.

**Main Points**

- The Creeping Fat Index (CFI) correlates with constrictive disease and may serve as a fast, simple, noninvasive prognostic marker.
- No relationship was found with visceral or subcutaneous fat, but median VSFR was significantly higher in stenotic patients.
- Ileocolonic involvement was associated with poor prognosis.
- Creeping Fat Index and ileocolonic disease may act as negative prognostic factors for early treatment planning.
- Identifying associations between Crohn’s disease, fat distribution, body mass index, obesity, and CFI could enable its clinical use as an imaging-based tool guiding early therapy and predicting progression.

## Introduction

**Figure 1.**
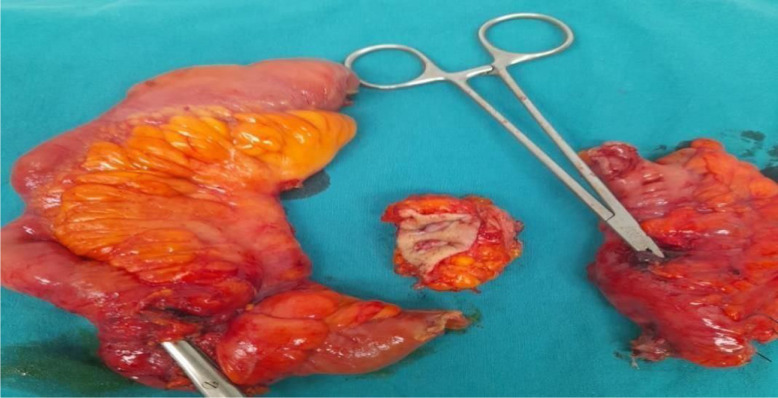
Macroscopic creeping fat appearance in surgical specimen.

**Figure 2.**
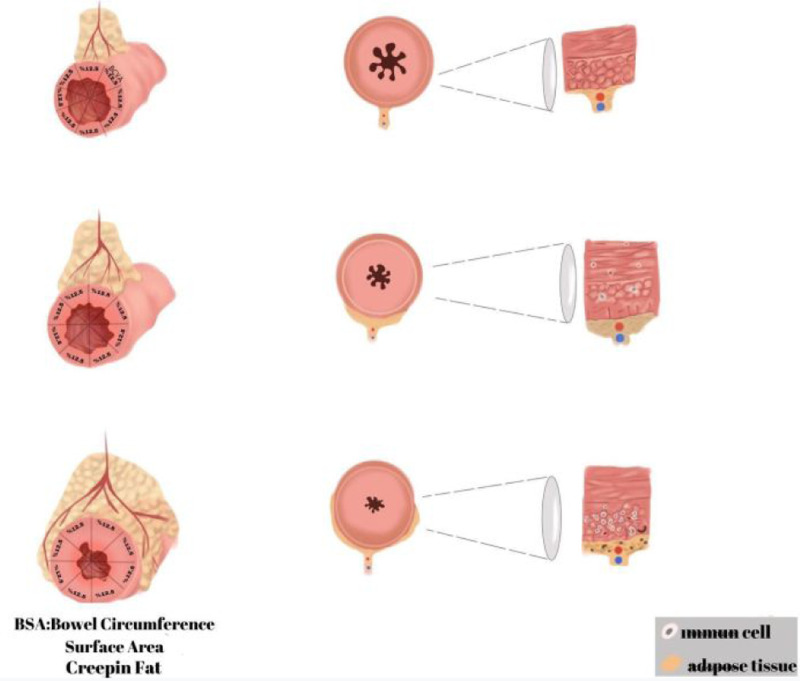
Relationship between creeping fat. (BSA [Bowel Surface Area] percentage and luminal stenosis in Crohn’s disease).

**Figure 3.**
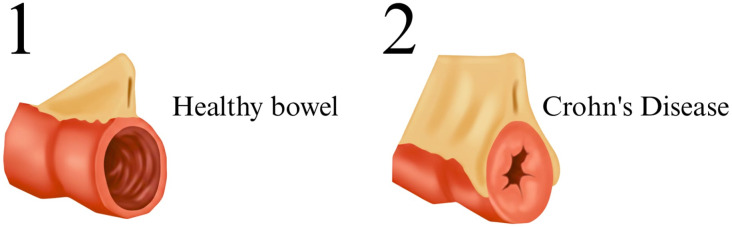
Creeping fat appearance in Crohn’s disease.

**Figure 4.**
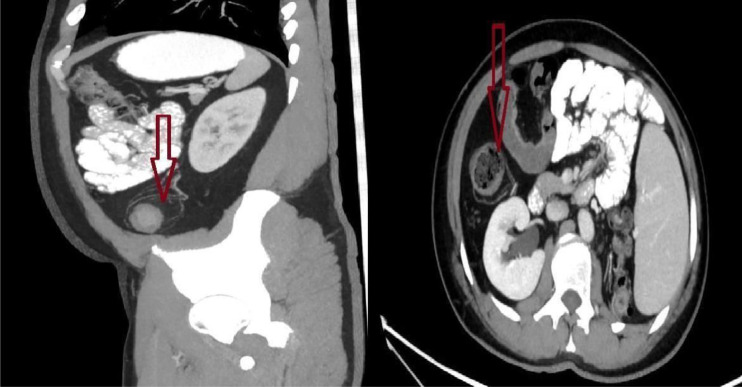
Creeping fat appearances in the sagittal and axial sections of computed tomography.

Creeping fat (CF) in Crohn’s disease (CD) patients refers to pathologically altered mesenteric adipose tissue around the inflamed portions of the intestine.^1^ (Figure 1) Hypertrophy of mesenteric adipose tissue is considered a consequence of inflammation, and it is thought that visceral adipose tissue plays an active role in the pathogenesis of the disease by producing proinflammatory cytokines.^2^ Recent studies have shown that CF may not only be a consequence of inflammation but also an active trigger of the fibrotic process. Long-chain free fatty acids secreted by CF (particularly palmitate) have been demonstrated to promote the proliferation of human intestinal smooth muscle cells, thereby contributing to muscularis propria thickening and stricture formation. These findings suggest that CF may play a pathophysiological role in stricture development in CD.^3^ (Figure 2, Figure 3) Establishing the relationship between CF and intestinal fibrostenosis, early prediction of stenosis formation, which forms the basis for surgical intervention in CD, and thus early initiation and/or escalation of medical treatment within the window of opportunity concept or relatively early decision to proceed with surgery may allow one to change the course of the disease. The aim of this study was to evaluate the relationship between the CF and visceral-subcutaneous fat findings evaluated by computed tomography (CT) in operated and non-operated CD patients with and without stenosis and to investigate whether the CF finding and visceral-subcutaneous fat ratios (VSFRs) in CD can be prognostic indicators (Figure 4).

## Materials and Methods

Institutional Review Board Statement: This study was performed in line with the principles of the Declaration of Helsinki. Approval was granted by the Ethics Committee of Ankara City Hospital (Date: 15/12/2021/No: E1-21-2197) on December 15, 2021. İn this study, CD patients who applied to Ankara City Hospital –Ankara Yıldırım Beyazıt University Faculty of Medicine Gastroenterology and Internal Medicine clinics between February 18, 2019, and September 31, 2021, were retrospectively examined.Written informed consent was obtained from the patients who agreed to participate in the study.

### Patients and Study Design

In this retrospective cross-sectional study, the relationship between CF and visceral-subcutaneous fat and intestinal fibrostenosis in CD was investigated. The study was approved by the institutional ethical review board. A total of 128 CD patients aged 18-70 years with clinical, endoscopic, and histological diagnosis of CD who were admitted to the hospital between February 18, 2019, and September 30, 2021, and who underwent a CT scan at diagnosis or follow-up to be evaluated for purposes of diagnosis and search for complications were included. Among the collected patients, 14 patients were excluded from the study due to inadequate image quality in terms of CF. If magnetic resonance was used as the imaging study, these patients were also excluded due to suboptimal scoring. 114 patients were finally included for analyses. Patients were grouped as follows: 31 patients with CD who had undergone bowel resection due to stenosis or obstruction, 39 patients with CD who had not yet undergone surgery for stenosis or obstruction but had stenosis, and 44 patients with CD who had no history of bowel resection or stenosis. Patients aged under 18, patients whose data could not be reached, pregnant patients, and those with gastrointestinal system malignancy were not included in the study. Patients were retrospectively examined in 3 groups through the hospital system and the online national health system of the Ministry of Health, and CF and visceral-subcutaneous fat findings evaluated by CT imaging method were compared with other groups. Among 31 operated patients, considering the possible negative effects of postoperative images on the measurements, patients with available computed tomographic imaging within 4 months before the operation were included in the study.

### Laboratory Parameters and Radiologic Evaluation

Demographic findings, disease-related characteristics, laboratory values closest to the date of CT, body mass index (BMI), CF index, CF density measurement, and visceral fat and subcutaneous fat measurements were extracted retrospectively. Creeping fat measurement was based on the bowel segment with stenosis. The present study employed the following criteria to establish a clear definition of intestinal stenosis: While defining intestinal stricture, localized lumen narrowing (at least a 50% reduction in lumen diameter measured compared to the normal adjacent bowel segment) and bowel wall thickening (a 25% increase in wall thickness compared to the adjacent unaffected bowel) were taken into account. The assessment of CF coverage was conducted in terms of percentages relative to the bowel surface area (BSACR).

### Computed Tomography Image Analysis

The CT images were reviewed by a radiologist with 18 years of experience in the field, with particular expertise and interest in IBD (Inflammatory Bowel Disease). To assess interrater reliability, a second radiologist with 10 years of experience in gastrointestinal radiology independently evaluated the scans, ensuring a reliable assessment of CF involvement.

The CT images of all patients were completed by evaluating the outpatient admission and request dates, and all examinations were performed on an elective basis. In addition, a review of the physicians’ notes revealed that a standard bowel preparation and contrast protocol had been applied.

The CT-based CF index used in this study is a semi-quantitative method that evaluates the percentage of mesenteric vascular extension around the intestine, similar to the Mesenteric CF Index (MCFI) described in a previous study.^4^ Since both methods rely on multiplanar reconstruction and the distribution of vascular structures, they are considered largely independent of inter-scanner technical variations. The cut-off values used in this study were determined based on the distribution observed in retrospective data analysis and were categorized to assess the statistical significance of the analyses. As all CT examinations were performed in the same center, using standardized protocols and the same brand/model scanner, inter-scanner variability and protocol-related measurement bias were minimized.

### Creeping Fat Density and Creeping Fat Index Bowel Surface Area Coverage Assessment:

Creeping fat was distinguished from other mesenteric fat alterations on CT images by its characteristic encasement of the intestinal wall together with mesenteric vessels. While changes such as inflammation and edema appear as more diffuse alterations in density, CF presents as a more localized, symmetric, and vessel-associated encasing pattern. After identifying the target bowel segment with CF on CT scans, a radiologic index based on the extent of the bowel circumference covered by the vascular structures within the adipose tissue was used to grade the fat content. According to this index, the circumference of the target bowel segment on axial and multiplanar-reformatted CT images was divided into 8 equal regions, and 12.5% was given for each region where mesenteric vessels overlapped. In addition, Hounsfield Unit (HU) measurement was performed by placing a “region of interest (ROI)” in the “CF” area surrounding the target intestinal segment. Creeping fat density was measured on 1.25 mm reconstructed images using the Hounsfield curve and standard attenuation values (air: −1000 HU, fat: −100 HU, water: 0 HU, iodinated contrast medium: +100 HU, bone: +1000 HU). To ensure consistency, soft tissue window settings were applied. The ROI was manually placed in the CF area surrounding the target bowel segment, with vascular structures and imaging artifacts carefully excluded.

### Visceral and Subcutaneous Fat Area Ratio:

Using software on the workstation, the visceral and subcutaneous fat areas of the patients included in the study were measured at the L3 vertebral level, and the visceral-subcutaneous fat area ratio was calculated. All these data were compared between 3 groups representing different prognostic behaviors.

### Statistical Analysis

Statistical analyses were performed using IBM SPSS Statistics for Windows, version 20.0 (IBM SPSS Corp.; Armonk, NY, USA). Normality was assessed with the Shapiro–Wilk test. Data are expressed as mean ± SD for normally distributed variables and as median (25%-75% quartiles) for non-normally distributed variables. Categorical variables are presented as numbers and percentages. Comparisons between 2 groups were conducted using the Student’s *t*-test or Mann–Whitney *U*-test, depending on distribution. For comparisons among 3 or more groups, ANOVA with Bonferroni test or Kruskal–Wallis with Dunn’s test was applied. Categorical data were analyzed with the chi-square test, Yates correction, or Fisher’s exact test. Correlations between numerical variables were assessed with Spearman analysis. Multivariate logistic regression, including variables associated with stenosis, identified independent predictors. A *P*-value < .05 was considered statistically significant. In 20 patients, CF density could not be measured due to poor CT quality or absent CF, and these cases were excluded; no imputation methods were used.

## Results

A total of 114 CD patients were included: 31 with bowel resection for stenosis/obstruction, 39 with non-operated stenosis, and 44 without stenosis or surgery. Demographic and clinical features are shown in. The male ratio was higher in the surgery group versus both non-operated stenosis and non-stenosis groups, and also in non-operated stenosis versus non-stenosis (*P* = .003). Surgery patients had lower hemoglobin, hematocrit, and albumin, but higher platelet, C-reactive protein (CRP), and sedimentation levels (Table 1).

The median CF density was −70 in non-operated patients, −90 in non-operated stenosis patients, and −55 in operated patients (*P* = .001). Subcutaneous fat levels were also lower in operated patients (−12 462) compared with non-operated groups (−17 755.5 and −17 872; *P* = .010). No difference was found between non-operated patients with and without stenosis. The VSFR was lower in patients without stenosis (0.4) than in those with non-operated stenosis (0.7) or operated patients (0.6; *P* = .011) (Table 1).

When the groups were dichotomized as those with stenosis and those without, VSFR and CRP were higher in those with stenosis. Stenosis patients were predominantly male and had lower albumin, whereas other demographic and laboratory findings were similar. Creeping fat density showed a weak positive correlation with CRP (*r* = 0.339, *P* = .001) and ESR (erythrocyte sedimentation rate) (r = 0.313, *P* = .002). Ileocolonic involvement was more frequent in stenosis patients, while ileal involvement was frequent in those without stenosis (Table 2).

The rates of patients with CF indexes of ≥50% were higher in the operated group and the non-operated group with stenosis compared to the group without stenosis (Table 3).

Compared to those without stenosis, those with stenosis had a CF involvement index of 37.5% (27.1% vs 18.2%; *P* < .001) and above (Table 4).

The multivariable regression model, including variables associated with stenosis, VSFR, CDAI, and CF index, was found to be independent predictors of stenosis (Table 5).

The rate of the CF involvement index of 37.5% and above was higher in ileocolonic patients compared to ileal patients (Table 6).

Finally, an excellent agreement was observed between the 2 assessments performed by the same observer at different times (ICC [Intraclass Corelation Coefficient] (3,1) = 0.968, 95% CI: 0.911-0.989, *P* < .001). The reliability further increased when the average of the measurements was considered (ICC (3,2) = 0.984, 95% CI: 0.953-0.995).

In the evaluations between 2 independent observers, a good level of agreement was found (ICC (3,1) = 0.781, 95% CI: 0.461-0.921, *P* < .001), which improved to a very good level when the average of the measurements was taken into account (ICC (3,2) = 0.877, 95% CI: 0.631-0.959).

## Discussion

The relationship between CD activity and BMI remains inconsistent. In a retrospective study, Blain et al^5^ (2002) reported that obesity was not linked to disease location or behavior, yet obese patients had higher risks of activity and hospitalization. Conversely, data from large US IBD cohorts showed that higher BMI did not worsen outcomes; in fact, overweight patients had better clinical results than those with normal weight.^6^ A 2017 meta-analysis also suggested obesity might be associated with a milder IBD course.^7^ More recently, Rudnicki et al^8^ found that morbid obesity in CD patients undergoing ileocolic resection correlated with increased complications and reoperation, though not statistically significant. Similarly, this study identified no significant association between CD and obesity. Some reports suggest childhood or adolescent obesity may increase IBD risk, though findings remain conflicting.^9^ Retrospective design, small sample size, nutritional status, incomplete steroid data, and environmental factors may have limited the ability to confirm such links. Traditional anthropometric measures, such as BMI and waist-to-hip ratio, are insufficient, whereas CT-based assessments of visceral fat provide more reliable prognostic insights in IBD.

Visceral-subcutaneous fat ratio at L3 did not show a significant association with stenosis (*P* = .058). Although close to significance, the limited sample size should be discussed in terms of this issue. Based on these results, VSFR cannot be regarded as a reliable standalone marker. Larger studies are required to clarify its prognostic potential.

Data from the literature support these findings. Büning et al^10^ reported that women with CD had higher body fat percentage, lower lean body mass, and increased visceral fat ratios in stricturing and penetrating cases.^4^ Prospective studies showed that the VAT/SAT (Visceral adipose tissue/Subcutaneous adipose tissue) ratio correlates with stricturing disease but not directly with surgery or hospitalization.^11^ Other studies linked visceral fat volume to penetrating disease and surgery.^12^

Creeping fat density did not differ significantly between patients with and without stricture, though measurement limitations may have affected reliability. The positive correlation between CF density and inflammatory markers suggests a link with inflammation. In CD, CF develops through immune cell infiltration, gradually encasing the bowel. Liu et al^13^ (2024) reported that long-chain free fatty acids secreted by CF stimulate intestinal smooth muscle proliferation, contributing to stricture formation.^13^ Thus, adipose tissue may initially protect but later promote muscular thickening with detrimental effects.^14^^,^^15^

Recently, CT-based indices have been developed, demonstrating accurate reflection of mesenteric fat wrapping in surgical specimens associated with fibrostenosis.^4^ The MCFI, defined by Li et al,^4^ is a CT-based scoring system that correlates strongly with fat wrapping in surgical samples and has proven accuracy in distinguishing moderate-to-severe fibrostenosis. In this study, BSACR, a percentage-based and more objective measure of the extent of fat coverage around the bowel, was used. Importantly, BSACR showed a significant association with stricture presence and disease prognosis, supporting its predictive value in clinical practice.^4^ Similarly, Althoff et al^16^ reported that CF detected by magnetic resonance imaging (MRI) was associated with complications and surgery. Despite methodological differences, these findings also highlight the prognostic potential of BSACR. Its quantitative and graded structure may further allow the determination of more precise thresholds to guide treatment decisions.^16^

The high complication and morbidity rates of CD highlight the need for early markers to predict severity. In this study, the percentage of CF surrounding the bowel on CT was calculated, and a CF index similar to previously defined mesenteric fat indices was used. Althoff et al^16^ reported that CF detected by MRI in 90 patients was associated with an increased risk of complications. More recent studies have shown that the MCFI is significantly associated with bowel wall thickening, mesenteric hypervascularity, fat stranding, abscess/fistula, and mesenteric lymphadenopathy, as well as with early postoperative recurrence.^17^ Furthermore, Li Xue et al^4^ demonstrated a strong correlation between MCFI and CF prevalence in surgical specimens and reported that the index had moderate accuracy in distinguishing moderate-to-severe fibrostenosis.

In this study, higher CF index values were associated with strictures, particularly above certain thresholds, suggesting a strong risk for stricture development. Higher CF index values were also more frequent in ileocolonic, penetrating, and stricturing cases, whereas low or absent CF involvement correlated with a more favorable disease course.

In conclusion, both the CF index and BSACR may represent adverse prognostic markers in CD, though larger prospective studies are needed to validate their utility.

### Limitations

The limited sample size is a common restriction in studies on CD, and increasing the sample size through multicenter studies may strengthen the results. The single-center, retrospective design also posed challenges in data interpretation and patient classification. Another limitation is the reliance solely on CT data; only patients who underwent imaging for suspected complications were included. Although BMI was not incorporated into multivariable regression due to lack of statistical significance, its known effects on fat distribution and inflammation make its exclusion a limitation. The use of a single evaluator in the primary analysis, validation of this assessment both internally and externally, and the inclusion of a relatively large patient cohort compared with the literature constitute the strengths of this study.

In this study, CF density could not be measured in 20 patients due to poor image quality. These patients were excluded, introducing potential selection bias. To minimize the influence of postoperative changes, only CT images obtained within 4 months before surgery were included. While this interval was considered appropriate for capturing active inflammation and preoperative status, it may have led to heterogeneity in timing and treatment responses.

Unmeasured variables such as steroid use, nutritional status, and disease duration may also have influenced CF development and stricture progression, highlighting the need for future studies to include these factors. Furthermore, the lack of sufficient evidence on the validity and reproducibility of the CF index and the absence of comparison with a gold standard remain important limitations. Prospective, multicenter, multiobserver studies are necessary to confirm the index’s reliability.

Despite these limitations, the study has strengths. The primary analysis relied on a single evaluator, ensuring methodological consistency, and both internal and external validity analyses were conducted. All radiological evaluations were performed by a radiologist with 18 years of IBD experience, minimizing interobserver variability. Moreover, the relatively large number of patients compared with previous studies strengthens the contribution of these findings to the existing literature.

The CF index defined in this study demonstrated a strong association with stricture development in CD. Computed tomography–based assessment of CF, being noninvasive, readily accessible, and rapidly applicable, offers practical advantages for clinical practice. Quantitative evaluation of the CF index on CT, particularly through its correlation with cases requiring surgery, carries the potential to predict the fibrostenotic course of CD at an early stage. In this respect, it may contribute to the identification of early treatment strategies aimed at preventing disease progression and enabling individualized patient follow-up.

The BSACR method used in this study provides a quantitative and objective measurement of CF extension on CT images by expressing it as a percentage, making it an important tool that could be integrated into clinical decision-making. The BSACR threshold values identified for stricture development (>37.5% and >50%) may assist in planning interventions such as treatment intensification (e.g., transition to immunosuppressive or biological agents) or early surgical consultation. In the future, such indices may be routinely employed as prognostic markers in treatment planning.

## Figures and Tables

**Table 1.** Demographic and Laboratory Findings According to Crohn’s Disease Phenotypes

Variables		CD Features			*P*
		Without Stenosis n = 44	Non-Operated Stenosis n = 39	Operated Patients n = 31	
Age, years		41 ± 14.7	41.6 ± 12.5	39.2 ± 10.2	.715
Gender, n (%)	Female	28 (63.6)	14 (35.9)	8 (25.8)	.003
	Male	16 (36.4)	25 (64.1)	23 (74.2)	
Comorbidities, n (%)		17 (38.6)	14 (35.9)	12 (38.7)	.969
Age at diagnosis, years, n (%)	>60	5 (11.4)	2 (5.1)	0	.367
	40-60	11 (25.0)	8 (20.5)	8 (25.8)	
	<40	28 (63.6)	29 (74.4)	23 (74.2)	
Smoker, n (%)	No	17 (38.6)	11 (28.2)	7 (22.6)	.522
	Quit	11 (25.0)	13 (33.3)	13 (41.9)	
	Yes	16 (36.4)	15 (38.5)	11 (35.5)	
BMI, kg/m^2^		24.9 ± 4.7	25.5 ± 3	24.0 ± 4.1	.326
CF density, HU		−70 (−88.5 to −53.5)	−90 (−98 to −72)	−55 (−84 to −45)	.001
Visceral fat, mm^2^		8554.5 (4613.25-12928.25)	12525 (7105-19479)	10635 (4107-14438)	.058
Subcutaneous fat, mm^2^		19904.5 (14998.75-29283)	17872 (14504-24241)	12462 (8272-18980)	.010
VSFR		0.4 (0.2-0.6)	0.7 (0.3-0.9)	0.6 (0.5-1.1)	.011
Laboratory findings					
WBC, 10^9^/L		7.8 (6.0-9.8)	8.7 (6.8-10.3)	8.4 (6.9-11.1)	.375
HB, g/dL		13.6 ± 2	13.6 ± 2.2	12.4 ± 1.9	.023
HTC, %		40.6 ± 5.6	41.5 ± 5.9	37.8 ± 5.7	.024
PLT, 10^9^/L		302 (233.75-386.75)	286 (229-379)	377 (262-523)	.012
CRP, mg/L		16 (2.0-37.4)	8.5 (3.1-20.7)	27.8 (11-68)	.001
Sedimentation, mm/hour		19 (6-34)	14 (5-20)	24 (12-47)	.013
Albumin, g/L		44.7 ± 4.4	44.0 ± 3.1	40.3 ± 5.1	<.001

Bonferroni correction was used for subgroup analysis.

BMI, body mass index; CD, Crohn’s disease; CF, creeping fat; CRP, C-reactive protein; HB, hemoglobin; HTC, hematocrit; HU, Hounsfield unit; PLT, platelet; VSFR, visceral fat/subcutaneous fat ratio; WBC, white blood cell.

**Table 2.** Distribution of Demographic and Laboratory Findings According to the Presence of a History of Stenosis

Variables		Without Stenosis n = 44	With Stenosis n = 70	*P*
Age, years		41 ± 14.7	40.5 ± 11.5	.834
Gender, n (%)	Female	28 (63.6)	22 (31.4)	<.001
	Male	16 (36.4)	48 (68.6)	
Comorbidities, n (%)		17 (38.6)	26 (37.1)	.999
Age at diagnosis, years, n (%)	>60	5 (11.4)	2 (2.9)	.164
	40-60	11 (25.0)	16 (22.9)	
	<40	28 (63.6)	52 (74.3)	
Smoker (%)	No	17 (38.6)	18 (25.7)	.252
	Quit	11 (25.0)	26 (37.1)	
	Yes	16 (36.4)	26 (37.1)	
BMI, kg/m^2^		24.9 ± 4.7	24.8 ± 3.6	.901
CF density, HU		−70.0 (−88.5 to −53.5)	−78.0 (−96.0 to −53.25)	.420
Visceral fat, mm^2^		8554.5 (4613.25-12928.25)	11573.5 (5066.0-19096.75)	.087
Subcutaneous fat, mm^2^		19904.5 (14998.75-29283.0)	16537.5 (10758.25-25551.25)	.225
VSFR		0.4 (0.2-0.6)	0.7 (0.4-0.9)	.003
Extension of involvement, n (%)	Ileal	26 (59.1)	23 (32.9)	.001
	Ileocolonic	16 (36.4)	47 (67.1)	
	Colonic	2 (4.5)	0	
Laboratory findings				
WBC, 10^9^/L		7.8 (6.2-9.8)	8.5 (6.8-10.6)	.169
HB, g/dL		13.6 ± 2.0	13.1 ± 2.1	.189
HTC, %		40.6 ± 5.6	39.9 ± 6.0	.509
PLT, 10^9^/L		296 (232-347.75)	318 (249-416)	.131
CRP, mg/L		4.6 (1.0-28.4)	14.3 (4.2-38.3)	.018
Sedimentation, mm/hour		13.5 (5.25-24)	17 (6-25)	.334
Albumin, g/L		44.7 ± 4.4	42.4 ± 4.5	.008

BMI, body mass index; CF, creeping fat; CRP, C-reactive protein; HB, hemoglobin; HTC, hematocrit; HU, Hounsfield unit; PLT, platelet; VSFR, visceral fat/subcutaneous fat ratio; WBC, white blood cell.

**Table 3.** Distribution of Clinical Scores According to Crohn’s Disease Phenotypes

		CD Features			
Variables		Without Stenosis n = 44	Non-Operated, with Stenosis n = 39	Operated n = 31	*P*
CDAI, n (%)	0-149	36 (81.8)	9 (23.1)	0 (0)	<.001
	150-220	6 (13.6)	21 (53.8)	3 (9.7)	
	221-450	2 (4.5)	9 (23.1)	28 (90.3)	
CF index, n (%)	No involvement/BSACR ≤ 25%	34 (77.3)	10 (25.6)	3 (9.7)	<.001
	BSACR 37.5%	8 (18.2)	7 (17.9)	12 (38.7)	
	BSACR ≥ 50%	2 (4.5)	22 (56.4)	16 (51.6)	

Bonferroni correction was used for subgroup analysis.

BSACR, bowel surface area coverage ratio; CD, Crohn’s disease; CDAI, CD activity index; CF, creeping fat.

**Table 4.** Distribution of Clinical Scores According to the Presence of Stenosis

Variables		Without Stenosis n = 44	With Stenosis n = 70	*P*
CDAI, n (%)	0-149	36 (81.8)	9 (12.9)	<.001
	150-220	6 (13.6)	24 (34.3)	
	221-450	2 (4.5)	37 (52.9)	
CF index, n (%)	No involvement/BSACR ≤ 25%	34 (77.3)	13 (18.6)	<.001
	BSACR 37.5%	8 (18.2)	19 (27.1)	
	BSACR ≥ 50%	2 (4.5)	38 (54.3)	

The stenosis rates of each BSACR group are statistically different from one another. For CDAI, the difference originates from the first group. Bonferroni correction was used for subgroup analysis.

BSACR, bowel surface area coverage ratio; CDAI, Crohn’s disease activity index; CF, creeping fat.

**Table 5.** Independent Predictors of Stenosis

Variables		Univariate			Multivariate		
		OR	95% CI	*P*	OR	95% CI	*P*
Gender, n (%)	Female	Reference					
	Male	3.82	1.72-8.46	.001			
VSFR		4.18	1.44-12.11	.008	23.79	3.64-155.58	.001
CRP, mg/L		1.04	1.01-1.08	.037			
Albumin, g/L		0.88	0.80-0.97	.010			
Disease duration, years		1.15	1.03-1.28	.012			
Perianal disease		3	1.22-7.40	.017			
Treatment, n (%)	Without medication	Reference					
	Classical treatments	1.82	0.40-8.25	.438			
	Biological agents	5.88	1.31-26.27	.020			
CDAI, n (%)	0-149	Reference					
	150-220	16	5.04-50.78	<.001	32.80	4.97-216.48	<.001
	221-450	74	14.95-366.31	<.001	190.38	19.02-1906.06	<.001
CF uptake index	No involvement	Reference					
	BSACR 37.5%	6.21	2.18-17.65	.001	12.53	1.86-84.45	.009
	BSACR% ≥ 50%	49.7	10.45-236.23	<.001	133.25	11.52-1540.80	<.001

BSACR, bowel surface area coverage ratio; CDAI, Crohn’s disease activity index; CF, creeping fat; CRP, C-reactive protein; OR, odds ratio, VSFR, visceral fat/subcutaneous fat rate.

**Table 6.** Distribution of Clinical Scores According to the Extent of Involvement

Extent of Involvement				
Variables		Ileal n = 49	Ileocolonic n = 63	*P*
CDAI, n (%)	0-149	28 (57.1)	16 (25.4)	
	150-220	14 (28.6)	15 (23.8)	<.001
	221-450	7 (14.3)	32 (50.8)	
CF involvement index, n (%)	No involvement/BSACR ≤ 25%	29 (59.2)	18 (27.7)	.001
	BSACR 37.5%	11 (22.4)	16 (24.6)	
	BSACR ≥ 50%	9 (18.4)	31 (47.7)	

The difference in BSACR groups arises from the difference between the ≤25% and ≥50% groups. For CDAI, the difference originates from the third group. Bonferroni correction was used for subgroup analysis.

BSACR, bowel surface area coverage ratio; CDAI, Crohn’s disease activity index; CF, creeping fat.

## Data Availability

The data that support the findings of this study are not openly available due to reasons of sensitivity and are available from the corresponding author upon reasonable request.
